# Thymoquinone in Atherosclerosis: A Multi-Target Nutraceutical Modulating Inflammation, Oxidative Stress, and Lipid Metabolism

**DOI:** 10.3390/nu18091480

**Published:** 2026-05-06

**Authors:** Weronika Fic, Karolina Kwaśniewska, Ewelina Polak-Szczybyło

**Affiliations:** 1 Student Scientific Club of Human Nutrition, Faculty of Health Sciences and Psychology, Collegium Medicum, University of Rzeszów, ul. Warzywna 1a, 35-959 Rzeszow, Poland; wf130811@stud.ur.edu.pl (W.F.); kk130958@stud.ur.edu.pl (K.K.); 2 Department of Dietetics, Faculty of Health Sciences and Psychology, Collegium Medicum, University of Rzeszów, ul. Warzywna 1a, 35-959 Rzeszow, Poland

**Keywords:** thymoquinone, *Nigella sativa*, atherosclerosis, inflammation, oxidative stress, lipid metabolism, nutraceuticals, cardiovascular disease

## Abstract

Background: Atherosclerosis is a chronic inflammatory disease driven by complex interactions between lipid metabolism disorders, oxidative stress, and immune dysregulation. Despite advances in pharmacotherapy, there is growing interest in nutraceutical compounds with multi-target effects. Thymoquinone (TQ), the main bioactive constituent of *Nigella sativa*, has emerged as a promising candidate due to its anti-inflammatory, antioxidant, and lipid-modulating properties. This review aims to comprehensively evaluate the effects of TQ on the key pathophysiological mechanisms involved in atherosclerosis, with particular emphasis on inflammation, oxidative stress, and lipid metabolism. Methods: A narrative review of preclinical studies, including in vitro and in vivo experimental models, was conducted to assess the biological activity of TQ and its potential anti-atherosclerotic effects. Results: TQ exhibits multi-target activity by modulating several molecular pathways associated with atherogenesis. It reduces oxidative stress by enhancing antioxidant enzyme activity and decreasing reactive oxygen species production. TQ also suppresses inflammatory signaling pathways, including NF-κB, MAPK, and COX-2, leading to decreased expression of pro-inflammatory cytokines such as IL-1β, IL-6, and TNF-α. Furthermore, it influences lipid metabolism by lowering total cholesterol and LDL-C levels while improving lipid profiles. TQ has also been shown to inhibit foam cell formation, endothelial dysfunction, and vascular inflammation. Additionally, nanocarrier-based formulations of TQ may improve its bioavailability and therapeutic potential. Conclusions: Current preclinical evidence suggests that TQ may play a significant role in the prevention and modulation of atherosclerosis through its multi-mechanistic action. However, the lack of well-designed clinical trials, limited bioavailability, and insufficient data on long-term safety highlight the need for further research to establish its clinical efficacy and optimal therapeutic use.

## 1. Introduction

In recent years, a significant increase in the prevalence of chronic cardiovascular diseases (CVD) has been reported in both developed and developing countries. The main underlying cause of this phenomenon is atherosclerosis, a chronic inflammatory disease of the arteries resulting from lipid accumulation and metabolic disturbances [1]. In the etiology of atherosclerosis, lifestyle is the primary determinant, including dietary patterns, physical activity, smoking [2], and chronic psychological stress [3]. Additionally, the risk of disease development is significantly increased by comorbidities such as obesity, type 2 diabetes, and metabolic syndrome [2]. The pathophysiology of atherosclerosis is particularly driven by the interplay between inflammation, oxidative stress, and lipid metabolism disorders [3]. Atherogenesis is initiated by endothelial dysfunction, which promotes the infiltration of low-density lipoproteins (LDL) into the vascular wall and their subsequent oxidative modification. Oxidized LDL, in synergy with pro-inflammatory cytokines, induces endothelial activation. Subsequently, monocytes migrate into the vessel wall and differentiate into macrophages, which engulf oxidized LDL, leading to the formation of foam cells and fatty streaks. Macrophage death, impaired efferocytosis, and chronic inflammation contribute to the expansion of the necrotic core, which is covered and stabilized by a fibrous cap. Excessive enlargement of the necrotic core relative to the thickness of the fibrous cap increases the risk of plaque rupture and thrombus formation [4]. The pathological processes occurring within atherosclerotic plaques account for a substantial proportion of CVD-related mortality worldwide. According to the World Health Organization (WHO), 19.8 million deaths were attributed to CVD in 2022, of which 85% were caused by myocardial infarction and stroke resulting from atherosclerosis [5]. The consequences of atherosclerosis also extend to the economic burden, generating significant healthcare costs [5,6]. The growing economic burden of atherosclerosis highlights the urgent need to develop innovative strategies targeting a key indicator of oxidative stress and inflammation [2]. In response to these public health challenges, increasing attention has been directed toward phytotherapy as an alternative to synthetic pharmacological agents.

Among widely used medicinal plants, *Nigella sativa* has gained particular interest. Its therapeutic properties have been recognized for centuries and utilized in traditional Middle Eastern medicine [7]. The health-promoting effects of *Nigella sativa* are primarily attributed to the presence of thymoquinone (TQ), a bioactive compound exhibiting a wide range of therapeutic properties. These include anti-inflammatory, antioxidant, immunomodulatory, hypolipidemic, hypoglycemic, anticancer, cardioprotective, and neuroprotective effects, among others [7]. The properties of TQ may directly modulate key pathophysiological mechanisms of atherosclerosis. Therefore, we can theoretically consider TQ as a compound with significant preventive potential in the area of CVD. Despite the growing number of studies on TQ, the current literature largely focuses on individual aspects of its activity, such as the modulation of inflammation, oxidative stress, and lipid metabolism. However, these studies do not comprehensively address the interconnected and parallel mechanisms underlying the pathophysiology of atherosclerosis. The lack of studies integrating these mechanisms limits the accurate evaluation of TQ’s role in the development and progression of this disease. A better understanding of the multi-target effects of TQ in atherosclerosis, a disease characterized by both inflammatory and lipid-related processes, may provide valuable insights into future research directions and therapeutic strategies. Therefore, the aim of this study is to analyze the effects of TQ on the pathophysiological mechanisms of atherosclerosis, with particular emphasis on lipid metabolism and inflammatory processes.

## 2. Methods

This study was conducted as a narrative review, the aim of which was to critically analyze the available scientific evidence regarding the role of TQ in modulating processes associated with atherosclerosis development, with a particular focus on inflammation, oxidative stress, and lipid metabolism disorders. A narrative approach was chosen due to the heterogeneity of available studies, including in vitro, in vivo, and clinical studies, which differ in methodology, endpoints, and study design, making a systematic synthesis or meta-analysis inappropriate.

A literature search was conducted between January and April 2026 using electronic databases including PubMed/MEDLINE, Scopus, Elsevier, and Google Scholar. The search strategy was based on combinations of keywords in English, used individually and in combination with Boolean operators (“AND” and “OR”). The following search terms were used: thymoquinone, black cumin, atherosclerosis, oxidative stress, inflammation, lipid metabolism, cardiovascular disease, endothelial dysfunction, foam cell formation, NF-κB signaling, and NLRP3 inflammasome. Relevant studies published in English between 2015 and 2026 were included. Previous publications were also included, where necessary, to provide essential information on the pathophysiology of atherosclerosis or mechanisms of action of thymoquinone.

Studies were selected based on their relevance to the topic. Titles, abstracts, and full texts were reviewed to identify publications addressing the role of thymoquinone in inflammation, oxidative stress, lipid metabolism, and mechanisms related to atherosclerosis. Editorials, conference abstracts, letters to the editor, duplicate records, and studies not directly relevant to the topic were excluded.

Due to the narrative nature of the review, no formal systematic review protocol or meta-analysis was used. However, priority was given to peer-reviewed studies that had a clear methodology and were consistent with existing evidence.

## 3. Characteristics of Thymoquinone

### 3.1. Physicochemical Properties of Thymoquinone

**Figure 1:**
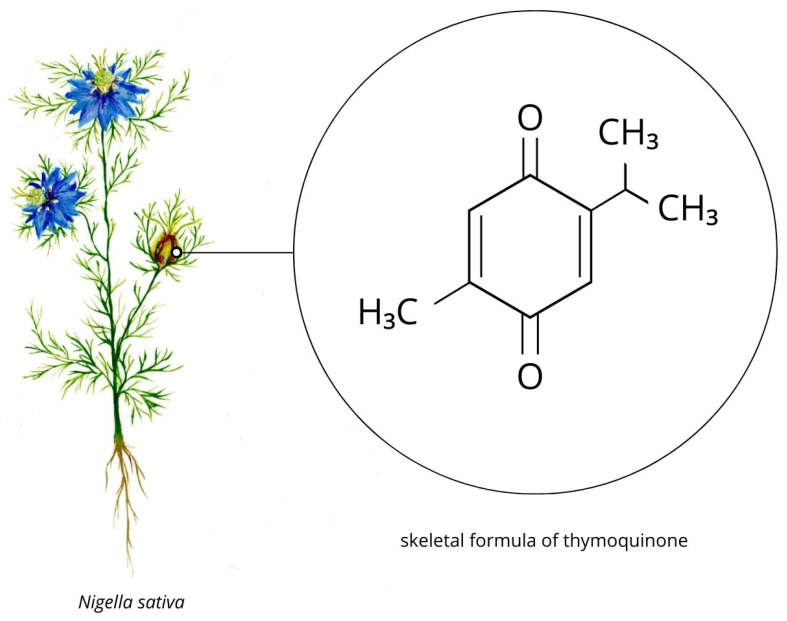
*Nigella sativa* as a natural source of TQ and the skeletal formula of TQ.

TQ is a biologically active constituent of the volatile oil derived from *Nigella sativa* seeds [7]. The concentration of TQ in black seed oil ranges from 18 to 25 μg/mL [8]. The compound was first isolated by El-Dakhakhny using thin-layer chromatography on silica gel [7]. From a chemical perspective, TQ is defined as 2-isopropyl-5-methylbenzo-1,4-quinone with the molecular formula C_10_H_12_O_2_ (Figure 1) [9]. TQ exists in tautomeric forms, including enol, keto, and mixed forms. The keto form is dominant, accounting for approximately 90%, and is primarily responsible for its pharmacological activity [7]. The molecular weight of TQ is 164.20 g·mol^−1^, its elimination half-life (T_1_/_2_) is approximately 217 min [9], and its melting point ranges between 49 and 50 °C [10]. TQ stability is enhanced under acidic conditions and is highly sensitive to light exposure [11]. Its aqueous solubility ranges from 549 to 669 mg/mL [8].

### 3.2. Pharmacokinetics of Thymoquinone

The bioavailability of compounds involves three main phases: availability for absorption in the intestinal lumen, absorption and retention, and distribution with subsequent incorporation into tissues and cells [12]. Due to its physicochemical properties, TQ exhibits low bioavailability, characterized by slow absorption and rapid metabolism [13]. In plasma, TQ binds spontaneously to albumin, which serves as a transport protein [7,12]. Protein binding exceeds 99% and does not affect protein secondary structure [13,14]. The lipophilic quinone moiety enables TQ to penetrate cellular and subcellular structures and interact with kinases and intracellular transcription factors [13]. Tekbaş et al. used GC-MS to evaluate serum TQ concentrations after oral administration of black cumin seed oil. Volunteers were orally administered black cumin oil containing 0.024 g and 0.072 g of TQ, respectively. The results of the analysis did not confirm the presence of TQ in human serum, which may be due to its physicochemical properties and the use of low doses of TQ [15]. The bioavailability of TQ can be improved by lipid-based nanocarriers, which enhance its solubility in aqueous environments, facilitate absorption across biological membranes, protect against degradation, and reduce rapid hepatic metabolism, thereby prolonging its activity and reducing toxicity [10,16]. However, these systems have limitations, including chemical and physical instability, lipid oxidation, and the requirement for high concentrations of surfactants, which may cause gastrointestinal irritation or toxicity at higher doses [10]. TQ is rapidly metabolized in the liver. Its main metabolic pathway is reduction to dihydrothymoquinone, which is responsible for its potent health-promoting effects [17]. Khalife et al. demonstrated that under physiological conditions, TQ undergoes intracellular non-enzymatic metabolic activation dependent on glutathione (GSH), nicotinamide adenine dinucleotide (NADH), or nicotinamide adenine dinucleotide phosphate (NADPH), leading to the formation of dihydrothymoquinone (DHTQ) [18]. In turn, the elimination of TQ metabolites involves their excretion in urine and feces [17].

## 4. Safety Profile of Thymoquinone

### 4.1. Pharmacological Interactions

Importantly, TQ exhibits significant metabolic interactions, including inhibition of cytochrome P450 enzymes [19,20]. Albassam et al. evaluated the effects of TQ on drug-metabolizing enzymes in human liver microsomes. Experimental systems included phenacetin with CYP1A2, tolbutamide with CYP2C9, dextromethorphan with CYP2D6, and testosterone with CYP3A4. TQ was tested at concentrations of 1, 10, and 100 μM, and metabolite levels were measured using high-performance liquid chromatography (HPLC). The results showed that TQ inhibited all tested enzymes, with the strongest inhibitory effects observed for CYP2C9, followed by CYP1A2, CYP3A4, and CYP2D6, indicating a potential for drug interactions [19].

Statins are used to treat hypercholesterolemia and are an important element of CVD prevention. They are metabolized by CYP450 enzymes, particularly the CYP3A4 isoform [21]. TQ, as an inhibitor of the CYP3A4 enzyme [19], may interfere with drug metabolism, potentially increasing the risk of serious clinical consequences [21]. However, there are studies confirming a synergistic interaction between TQ and simvastatin, a drug used in the treatment of hypercholesterolemia, but these studies concern only anticancer effects [22,23]. However, no studies confirming this phenomenon in atherosclerosis have been reported. Antihypertensive drugs such as angiotensin-converting enzyme inhibitors, beta-blockers, calcium channel blockers, and diuretics are metabolized by CYP50 [24], making them potential competitors for the active site of the enzyme with TQ [19]. As a result, their metabolism may be disrupted, which in turn may result in a reduced therapeutic effect or increased adverse effects. However, there are currently no clinical or preclinical studies confirming this hypothesis. TQ may potentially interact pharmacologically with antiplatelet drugs by affecting CYP450 enzymes [19] involved in the metabolism of these drugs [25]. However, studies confirming these interactions are lacking. Warfarin is a drug used in anticoagulant therapy and the prevention of thrombosis. This drug is primarily metabolized by CYP2C9. By binding to the active site of the enzyme, TQ inhibits its activity, leading to increased warfarin concentrations in blood and possible adverse effects [26]. The lack of clinical and preclinical studies explaining the mechanisms of potential interactions between TQ and drugs used in atherosclerosis prevents its inclusion as an adjunct to standard therapy.

### 4.2. Toxicity of Thymoquinone

The safety profile of TQ remains an important issue. The lethal dose (LD_50_) of TQ depends on the experimental model and route of administration. Toxicological studies indicate that LD_50_ values are higher following oral administration compared to intraperitoneal injection. The average LD_50_ in rats is approximately 790 mg/kg (oral) and 57 mg/kg (intraperitoneal), with observable signs of toxicity [13]. The no observed adverse effect level (NOAEL) in subacute toxicity studies is approximately 10 mg/kg [27]. Yazan et al. evaluated the acute toxicity of intravenous TQ-NLC at a dose of 25 mg/kg in rats. After a 14-day intervention, the results showed no significant changes in physical, hematological, biochemical, or histopathological parameters, except for local inflammation at the injection site [28]. In contrast, in the assessment of acute toxicity, it was demonstrated that TQ at the same dose caused 100% mortality, whereas TQ-NLC resulted in only a single death. Meanwhile, in the subacute toxicity study, no significant changes were observed in body weight, organ-to-body weight ratio, histology, or hematological and biochemical profiles [29]. In contrast, a randomized, double-blind, placebo-controlled phase I clinical trial in healthy individuals assessed the safety of black seed oil containing 5% TQ at a dose of 200 mg/day for 90 days. The study demonstrated no significant adverse effects or clinically relevant changes in renal and hepatic parameters, while also suggesting potential therapeutic benefits [30]. Current toxicological data are insufficient to meet regulatory requirements for the initiation of large-scale clinical trials [11]. All data regarding doses and duration of use come from animal studies. Differences in metabolic processes between humans and rodents prevent direct transfer of results between species [31]. Furthermore, the differential toxicological response of males and females in preclinical studies may suggest the need to adjust TQ doses in clinical trials, taking into account potential gender differences [32]. Furthermore, there is a lack of data regarding the long-term toxicity of TQ and a lack of analyses assessing the risk of TQ use in specific clinical groups. Due to the teratogenic effects of TQ observed in in vivo studies in animals, caution is warranted when using products containing this ingredient during the first weeks of pregnancy [33,34]. However, it should be emphasized that this effect has not yet been confirmed in clinical studies. At present, there is a lack of precise data to determine the maximum safe dose of TQ in humans. Nevertheless, available evidence suggests that TQ exhibits low toxicity and a relatively low risk of mortality. Further research is required to confirm its safety and establish optimal dosing strategies [11]. A summary of the physicochemical properties, pharmacokinetics, and safety profile of TQ is presented in Table 1.

## 5. Mechanisms of Thymoquinone in Atherosclerosis

Atherosclerosis is a chronic, progressive disease that develops in a sequence of interrelated pathophysiological stages, starting with endothelial dysfunction and lipid accumulation, through oxidative stress and persistent inflammation, to foam cell formation, progression and destabilization of atherosclerotic plaque, and finally thrombosis. These stages are closely interconnected through common molecular pathways, including increased generation of reactive oxygen species (ROS), activation of redox-sensitive transcription factors such as NF κB, and disruption of lipid metabolism and inflammatory mediators [4]. TQ, the main bioactive component of *Nigella sativa*, exhibits antioxidant, antidiabetic, and neuroprotective effects, and has a beneficial effect on cardiovascular parameters, as well as pronounced anti-inflammatory and lipid-lowering properties, which are important in the context of atherosclerosis [35,36,37,38,39,40]. TQ modulates the activity of antioxidant enzymes such as catalase, superoxide dismutase, and glutathione peroxidase, enhancing the endogenous defense system against oxidative stress and reducing the production of ROS. [41] Consequently, this limits the activation of NF κB and the secondary expression of inflammatory mediators [42,43]. TQ has been shown to reduce the concentrations of proinflammatory cytokines such as IL-1β, IL-6, TNF α, and IFN γ, inhibit COX 2 expression, and interfere with MAPK pathways, leading to a reduced inflammatory response in the cardiovascular system [38,39,41,42,43,44,45]. In various experimental models, TQ attenuates key atherogenic mechanisms. It improves endothelial function and nitric oxide (NO) bioavailability, inhibits the uptake of LDL-derived cholesterol and the expression of the LOX-1 receptor in endothelial cells and macrophages, reduces lipid peroxidation and malondialdehyde (MDA) levels, and decreases the expression of chemokines and adhesion molecules involved in leukocyte recruitment (MCP-1, ICAM-1, VCAM-1, E-selectin) [44,46,47,48,49,50,51,52]. Furthermore, TQ favorably modifies the lipid profile by lowering the concentrations of total cholesterol, LDL C, triglycerides and VLDL C, while maintaining or increasing the concentration of HDL C in models with a high-fat diet and in animals genetically predisposed to hyperlipidemia [45,52,53,54]. Through these pleiotropic effects, TQ may influence multiple stages of atherogenesis—from early endothelial damage to plaque progression and thrombosis. It should be emphasized, however, that most available studies focus on the effects of TQ on individual signaling pathways such as NFκB, MAPK, NLRP3, and LOX-1, analyzing them largely in isolation. Consequently, despite the extensive dataset available, these pathways are rarely integrated into a coherent molecular regulatory network encompassing the successive stages of atherosclerosis development.

### 5.1. Endothelial Dysfunction and Vascular Damage

Endothelial dysfunction is considered a key early event in atherogenesis and is characterized by impaired NO-dependent vasodilation, increased permeability to lipoproteins, loss of antithrombotic properties, and increased expression of adhesion molecules promoting leukocyte recruitment. Proinflammatory stimuli and oxidative stress activate NF-κB in endothelial cells, leading to increased expression of ICAM-1, VCAM-1, E-selectin, and chemokines, including MCP-1, which facilitate monocyte adhesion and migration to the vascular intima [46]. TQ demonstrates significant vasoprotective effects by improving endothelium-dependent vasodilation, normalizing nitric oxide synthase activity, and reducing ROS levels in the vessels of elderly individuals or those with impaired endothelial function. In human coronary artery endothelial cells (HCAEC), black seed oil and TQ reduced LPS-induced endothelial activation by inhibiting ICAM-1 and VCAM-1 expression at the mRNA and protein levels and by reducing monocyte adhesion; a stronger effect was observed at higher TQ concentrations. TQ also modulates the TNF-α–NF-κB signaling pathway and reduces the expression of adhesion molecules and chemokines (ICAM-1, MCP-1) in endothelial cell and THP-1 macrophage models, resulting in reduced leukocyte recruitment and early inflammatory activation of the endothelium. The collected data indicate that TQ may counteract endothelial dysfunction and the initial inflammatory stimulation preceding atherosclerotic plaque formation [47,48,49,50,51].

### 5.2. Lipid Metabolism and Lipid Accumulation

Following endothelial damage, increased permeability and retention of apoB-containing lipoproteins in the subendothelial space promote lipid accumulation and the formation of fatty streaks. Oxidized LDL fractions are more effectively captured by macrophage scavenger receptors, and the persistent proatherogenic lipid profile (increased total cholesterol, LDL-C, triglycerides, and VLDL-C with reduced HDL-C) accelerates plaque development. Numerous animal studies have demonstrated that TQ has a beneficial effect on lipid metabolism in models of hyperlipidemia and a high-fat diet. In ApoE−/− and LDL-R−/− mice fed a high-cholesterol diet, TQ supplementation led to a significant reduction in total cholesterol and LDL-C levels and to a reduction in the extent of atherosclerotic lesions and myocardial damage [45,53]. Idris-Khodja et al. hypothesized that chronic administration of TQ may restore vascular function. In Wistar rats aged 12–15 weeks (young) and 16–20 months (old), older animals received TQ in drinking water at doses of 10 or 30 mg/kg/day for 2–4 weeks. TQ improved endothelium-dependent vasodilation in a dose-dependent manner in old rats with impaired vascular relaxation. It also normalized nitric oxide synthase activity, reduced ROS levels, and decreased COX-1 and COX-2 expression in arteries, thereby preventing excessive vasoconstriction. These findings indicate a strong vasoprotective, antioxidant, and anti-inflammatory effect of TQ [51]. Ragheb et al. investigated whether TQ could inhibit atherosclerosis progression and/or improve lipid parameters and oxidative stress in male rabbits. The animals were divided into four groups: control, a group fed a diet containing 1% cholesterol, and two groups receiving the same diet supplemented with TQ at doses of 10 or 20 mg/kg/day. Blood samples were collected at baseline, 4 weeks, and 8 weeks. After 8 weeks, total cholesterol significantly increased in the cholesterol-only group, whereas it decreased significantly in both TQ-treated groups, although the higher TQ dose did not yield a proportionally greater reduction. Lower TC levels were considered one of the key factors reducing lipid accumulation in the aortic wall and slowing plaque development [52]. Nader et al. evaluated the effects of propolis and TQ, administered separately, on plaque formation, lipid profile, and inflammatory markers in rabbits fed a high-cholesterol diet. Both TQ and propolis significantly reduced total cholesterol and LDL-C and maintained higher HDL-C levels. In addition, the degree of atherosclerotic lesion development was markedly lower in treated groups, with cleaner vessels, less vascular wall damage, and fewer foam cells. The authors suggested that these substances might represent a useful strategy for the prevention of atherosclerosis and cardiovascular diseases [54]. Xiao et al. also examined the cardioprotective role of TQ in genetically susceptible mice fed a high-fat diet. TQ administration reduced total cholesterol and LDL-C, lowered inflammatory markers, decreased macrophage accumulation in cardiac tissue, improved cardiac function, and reduced histopathological damage [45]. Similar lipid-lowering effects were reported in high-fat diet-induced obese rats, in which TQ treatment reduced body weight gain and adipocyte size, improved hyperlipidemia, and normalized leptin and adiponectin levels. Supplementation significantly lowered TC, TG, LDL-C, and VLDL-C while increasing HDL-C [55].

### 5.3. Oxidative Stress and Redox Imbalance

Oxidative stress plays a central role in atherogenesis, promoting LDL oxidation, endothelial dysfunction, smooth muscle cell activation, and apoptosis of cells within the plaque. Excessive ROS production, often associated with the activity of NADPH oxidases and mitochondrial sources, leads to lipid peroxidation and the formation of reactive aldehydes such as MDA and 4HNE, which further exacerbate vascular damage and the inflammatory response. TQ is a potent antioxidant that enhances endogenous defense systems and directly reduces ROS production [40,41]. In various in vivo models, TQ increased the activity of superoxide dismutase, catalase, and glutathione peroxidase and decreased the concentration of MDA and other markers of lipid peroxidation, protecting vascular and cardiac tissues from oxidative damage [41,45,52,56,57,58]. In aging rats, chronic TQ administration led to a decrease in ROS levels, normalization of nitric oxide synthase function, and decreased COX-1/COX-2 expression, resulting in improved vascular reactivity [51]. In models of myocardial ischemia–reperfusion and diabetic cardiomyopathy, TQ prevented the depletion of antioxidant reserves, maintained normal levels of reduced glutathione, and limited oxidative damage to cardiomyocytes [56,57,58,59]. Through this potent antioxidant effect, TQ interferes with the oxidative modification of lipids and proteins, which is crucial for the subsequent stages of inflammatory activation and foam cell formation.

### 5.4. Inflammation and Pro-Inflammatory Pathways

Atherosclerosis is currently recognized as a chronic inflammatory disease in which both innate and adaptive immune responses drive the initiation and progression of atherosclerotic plaque. Cytokines such as TNF-α, IL-1β, IL-6, and IFN-γ, together with chemokines (e.g., MCP-1) and adhesion molecules (ICAM-1, VCAM-1, E-selectin), coordinate the recruitment and activation of inflammatory cells, while NF-κB and MAPK pathways integrate many of these signals. TQ exhibits broad anti-inflammatory activity by modulating these pathways at multiple levels. It inhibits NF-κB activation by reducing the phosphorylation and degradation of IκB, leading to decreased expression of pro-inflammatory cytokines, chemokines, and adhesion molecules in endothelial cells and macrophages [41,42,43,47,48,49]. In a model of LPS-stimulated endothelial cells, both Nigella sativa extract (NSE) and TQ reduced the expression of MCP-1, VEGF, NLRP3 inflammasome-related genes, and IL-1β. Additionally, NSE decreased IL-6 and IL-8 levels and increased TET2 expression, suggesting a potential epigenetic role in the regulation of inflammatory responses [48]. In LDLR−/− mice, TQ reduced serum hs-CRP levels and the expression of NLRP3, IL-1β, IL-18, IL-6, TNF-α, and caspase-1, which is consistent with the attenuation of vascular inflammation and pyroptosis [53]. These findings, supported by data from other inflammatory disease models, indicate that TQ modulates key nodes of inflammatory signaling pathways relevant to all stages of atherogenesis [38,39,40].

### 5.5. Foam Cell Formation and Plaque Progression

Foam cells are formed as a result of the intensive uptake of modified LDL particles by scavenger receptors expressed on monocytes/macrophages, followed by the accumulation of cholesterol esters, which leads to the formation of fatty streaks and the lipid core of advanced atherosclerotic plaques. Pro-inflammatory cytokines, such as TNF-α and IFN-γ, enhance the expression of scavenger receptors and impair cholesterol efflux from macrophages, thereby accelerating foam cell formation and lesion progression. TQ has been shown to inhibit foam cell formation by reducing the expression of LOX-1, a key receptor involved in the uptake of oxidized LDL (oxLDL) by endothelial cells and macrophages. In ApoE−/− mice, TQ decreased LOX-1 expression in the vascular wall, reduced macrophage infiltration within atherosclerotic lesions, and limited collagen deposition and myocardial fibrosis, suggesting attenuation of both plaque burden and cardiac remodeling [45]. Huwait et al. assessed the effects of TQ on human THP-1 macrophages in vitro. TQ did not affect cell viability but suppressed IFN-γ-induced ICAM-1 and MCP-1 gene expression and reduced monocyte migration toward MCP-1. No significant effect on cholesterol content in THP-1 macrophages was observed. Additionally, in silico analysis confirmed the therapeutic potential of TQ in lipid disorders and atherosclerosis [49]. However, the presented mechanisms should be treated as hypotheses supported by preclinical data that require further verification in translational and clinical studies.

### 5.6. Plaque Instability and Thrombosis

In advanced stages of atherosclerosis, plaques may become unstable due to the presence of a large necrotic core, a thin fibrous cap, persistent inflammation, and increased metalloproteinase activity, which predispose them to rupture and thrombus formation. Inflammatory mediators increase the expression of tissue factor (TF) and plasminogen activator inhibitor 1 (PAI 1), promoting a prothrombotic environment, while activated platelets and coagulation factors participate in the formation of an occlusive thrombus following plaque disruption. Although data on the direct effect of TQ on plaque stability and thrombosis are limited, several observations provide important mechanistic insights. In an in vitro study, TQ had minimal effects on standard coagulation parameters but attenuated the prothrombotic state induced by TNFα by modulating NFκB-dependent inflammatory and coagulation pathways [47]. In animal models of hypercholesterolemia and myocardial damage, TQ reduced fibroinflammatory remodeling, improved cardiac function, and limited histopathological damage, which may indirectly promote more stable vascular and myocardial morphology [45,52,54,56,57,58,59]. Although these studies do not directly address atherosclerosis, by simultaneously reducing oxidative stress and inflammation and improving the lipid profile, TQ likely reduces the proinflammatory and prothrombotic environment conducive to plaque destabilization and thrombus formation, although this hypothesis requires confirmation in dedicated models of plaque stability and thrombosis.

Table 2 provides a comprehensive overview of preclinical and clinical studies investigating the effects of TQ on key mechanisms involved in atherosclerosis, including oxidative stress, inflammation, lipid metabolism, and cardiovascular function.

## 6. Limitations and Research Gaps

Despite promising preclinical research results, knowledge about TQ is not fully systematized, and the available data indicate numerous limitations and insufficiently explored areas. The heterogeneity of current research, based on different experimental models, methodologies, and doses, hinders comprehensive analysis of results and drawing consistent conclusions. Furthermore, the insufficient number of studies on atherosclerosis models hinders a clear analysis of the disease’s pathomechanisms. Another problem is the lack of studies assessing the pharmacological interactions of TQ with drugs used in atherosclerosis. This prevents the determination of the safety of TQ use in clinical practice. TQ’s low bioavailability limits the possibility of reliably assessing its biological activity. Furthermore, there is a lack of clinical data confirming the efficacy and safety of nanoformulations. The pharmacokinetics of TQ are still poorly understood, which translates into a lack of toxicological data defining a safe dose of the compound. Information regarding the long-term safety of TQ use and the identification of groups at increased risk in humans is also lacking. The presence of so many unknowns means that there is currently no basis for conducting randomized clinical trials assessing the effectiveness of TQ in atherosclerosis. Further preclinical research in these areas is necessary to establish a strong foundation for initiating clinical trials.

## 7. Conclusions

Available preclinical studies confirm the multi-target anti-atherosclerotic effects of TQ, including the reduction in inflammation and oxidative stress, as well as the modulation of lipid metabolism and endothelial function. Despite these promising findings, the current body of evidence does not allow for a clear conclusion regarding the efficacy of TQ in improving atherosclerosis-related markers in humans. The insufficient number of preclinical studies on atherosclerosis models, the heterogeneity of available studies, and the lack of clinical trials examining the safety of TQ and its nanocarriers limit the development of randomized clinical trials. The lack of randomized clinical trials prevents a comprehensive analysis of TQ’s effect on atherosclerotic processes and the assessment of its future use both in prevention and as an adjunct to therapy. Given these limitations, it is not yet possible to unequivocally recommend TQ as a therapeutic agent for atherosclerosis. Further research is required, particularly well-designed clinical trials, to determine its efficacy, establish optimal dosing strategies, evaluate appropriate pharmaceutical formulations, and confirm its long-term safety in humans.

## Figures and Tables

**Table 1:** Physicochemical properties, pharmacokinetics, and safety profile of TQ.

Ref.	Category	Key Information
[9]	Chemical name	2-isopropyl-5-methylbenzo-1,4-quinone
[9]	Molecular formula	C_10_H_12_O_2_
[9,10,11]	Physicochemical properties	Molecular weight—164.20 g·mol^−1^ Half-life—approx. 217 min Melting point—49–50 °C Solubility in aqueous environment—549–669 mg/mL High stability at low pH Photosensitive molecule
[13]	Pharmacokinetics	Low bioavailability Slow absorption Rapid metabolism
[13,19,20,21,22,23,24,25,26,27,32,33]	Safety	Potential interactions with anti-atherosclerotic drugs LD_50_ ~790 mg/kg (oral) LD_50_ ~57 mg/kg (intraperitoneal) NOAEL ~10 mg/kg Teratogenicity
	Limitations	Lack of clinical studies Only preclinical studies available No standardized safe dose for humans

LD_5__0_—median lethal dose, NOAEL—no observed adverse effect level.

**Table 2:** Summary of preclinical and clinical studies evaluating the effects of TQ on atherosclerosis-related mechanisms, including oxidative stress, inflammation, lipid metabolism, and cardiovascular function.

Ref.	Study Model	TQ Dose/Concentration	Method of administration	Duration	Key Findings
[16]	Rats	20 mg/kg	Oral administration	48 h	Nanostructured lipid carriers (NLC) increased bioavailability 2.03–3.97-fold and prolonged half-life compared to TQ suspension.
[19]	Human liver microsomes	1, 10, 100 µM	In vitro	Short incubation	Inhibition of CYP450 enzymes (CYP2C9, CYP1A2, CYP3A4, CYP2D6), suggesting potential drug interactions.
[28]	Female rats (acute toxicity)	25 mg/kg (i.v., TQ-NLC)	Intravenous administration	14 days	No systemic toxicity observed; only local inflammation at injection site.
[29]	BALB/c mice (acute & subacute toxicity)	Acute: 5–300 mg/kg; Subacute: 1–100 mg/kg	Oral administration	14/28 days	Pure TQ showed higher toxicity than NLC; subacute doses up to 100 mg/kg were safe.
[30]	Humans (phase I clinical trial)	200 mg/day (5% TQ oil)	Oral administration	90 days	No adverse effects; no changes in renal and hepatic parameters; confirmed safety.
[45]	ApoE−/− mice (atherosclerosis model)	20 mg/kg (oral)	Oral administration	8 weeks	↓ LOX-1 expression, ↓ TC and LDL, ↓ macrophage accumulation, ↑ antioxidant enzymes.
[47]	In vitro	10–50 µM	In vitro	6–24 h	Minimal effect on coagulation; inhibition of TNF-α/NF-κB signaling.
[48]	Human endothelial cells	1–10 µM	In vitro	24 h	↓ MCP-1, VEGF, NLRP3, IL-1β; ↑ TET-2 expression.
[53]	LDL-R−/− mice (high-fat diet)	50 mg/kg (oral)	Oral administration	8 weeks	↓ TC, LDL-C, IL-1β, IL-6, TNF-α; inhibition of NLRP3 inflammasome.
[49]	THP-1 macrophages	5–20 µM	In vitro	24 h	↓ ICAM-1, MCP-1 expression and monocyte migration; no effect on viability.
[50]	HCAEC endothelial cells	4.5–36 µM	In vitro	24 h	↓ ICAM-1, VCAM-1 expression; reduced monocyte adhesion and endothelial activation.
[51]	Wistar rats (aging model)	10–30 mg/kg	Oral administration	2–4 weeks	Improved endothelial-dependent vasodilation; ↓ ROS; normalization of NO synthase.
[52]	Rabbits (1% cholesterol diet)	10–20 mg/kg	Oral administration	8 weeks	↓ total cholesterol; reduced lipid accumulation in aorta.
[54]	Rabbits (high-cholesterol diet)	10 mg/kg	Oral administration	8 weeks	↓ TC and LDL; ↑ HDL; reduced atherosclerotic lesions and foam cells.
[45]	Mice (genetic model)	20 mg/kg	Oral administration	8 weeks	↓ TC, LDL; ↓ inflammation; improved cardiac function.
[55]	Rats (diet-induced obesity)	50 mg/kg (oral)	Oral administration	4 weeks	↓ body weight, ↓ TG, ↓ LDL; ↑ HDL; normalization of leptin and adiponectin.
[59]	Isolated rat heart (Langendorff)	10–20 µM	Ex vivo perfusion	Acute (I/R model)	Cardioprotection; ↓ apoptosis (↓ Bax, ↑ Bcl-2); improved cardiac function.
[56]	Rats (STZ-induced diabetes)	50 mg/kg	Oral administration	4 weeks	↑ SOD and catalase; ↓ MDA; ↓ fibrosis and apoptosis.
[57]	Rats (isoproterenol injury)	20–50 mg/kg	Oral administration	14 days	Dose-dependent ↓ myocardial necrosis, ↓ inflammation and fibrosis.
[58]	Wistar rats (myocardial infarction)	20 mg/kg	Oral administration	21 days	Cardioprotection; ↓ lipid peroxidation and inflammatory cytokines.

TQ—thymoquinone; TC—total cholesterol; LDL—low-density lipoprotein; HDL—high-density lipoprotein; ROS—reactive oxygen species; NLC—nanostructured lipid carriers; MCP-1—monocyte chemoattractant protein-1; ICAM-1—intercellular adhesion molecule-1; VCAM-1—vascular cell adhesion molecule-1; ↑ increase; ↓ decrease.

## Data Availability

Not applicable.

## Abbreviations

The following abbreviations are used in this manuscript:

CVD	cardiovascular diseases
COX-2	cyclooxygenase-2
HCAEC	human coronary artery endothelial cells
HDL	high-density lipoprotein
HDL-C	high-density lipoprotein cholesterol
ICAM-1	intercellular adhesion molecule-1
IFN-γ	interferon gamma
IL	interleukin
LDL	low-density lipoprotein
LDL-C	low-density lipoprotein cholesterol
LOX-1	lectin-like oxidized low-density lipoprotein receptor-1
MAPK	mitogen-activated protein kinase
MCP-1	monocyte chemoattractant protein-1
MDA	malondialdehyde
NF-κB	nuclear factor kappa B
NLC	nanostructured lipid carriers
NLRP3	NLR family pyrin domain containing 3
NO	nitric oxide
NOAEL	no observed adverse effect level
NSE	*Nigella sativa* extract
PAI-1	plasminogen activator inhibitor-1
PEG	polyethylene glycol
ROS	reactive oxygen species
SOD	superoxide dismutase
STZ	streptozotocin
TC	total cholesterol
TF	tissue factor
TG	triglycerides
TNF-α	tumor necrosis factor alpha
TQ	thymoquinone
VLDL	very low-density lipoprotein
LD_5__0_	median lethal dose
